# Pre- and Post-Treatment with Novel Antiepileptic Drug Oxcarbazepine Exerts Neuroprotective Effect in the Hippocampus in a Gerbil Model of Transient Global Cerebral Ischemia

**DOI:** 10.3390/brainsci9100279

**Published:** 2019-10-17

**Authors:** Ji Hyeon Ahn, Bich Na Shin, Joon Ha Park, Tae-Kyeong Lee, Young Eun Park, Jae-Chul Lee, Go Eun Yang, Myoung Cheol Shin, Jun Hwi Cho, Kyu Chang Lee, Moo-Ho Won, Hyeyoung Kim

**Affiliations:** 1 Department of Biomedical Science, Research Institute of Bioscience and Biotechnology, Hallym University, Chuncheon, Gangwon 24252, Korea; jh-ahn@hallym.ac.kr; 2 Department of Physiology, School of Medicine, Hallym University, Chuncheon, Gangwon 24252, Korea; tlsqlsck21@nate.com; 3 Department of Anatomy, College of Korean Medicine, Dongguk University, Gyeongju, Gyeongbuk 38066, Korea; jh-park@dongguk.ac.kr; 4 Department of Neurobiology, School of Medicine, Kangwon National University, Chuncheon, Gangwon 24341, Korea; xorud312@naver.com (T.-K.L.); taeparo@naver.com (Y.E.P.); anajclee@kangwon.ac.kr (J.-C.L.); 5 Department of Radiology, Kangwon National University Hospital, Chuncheon, Gangwon 24289, Korea; yangke@kangwon.ac.kr; 6 Department of Emergency Medicine, School of Medicine, Kangwon National University, Chuncheon, Gangwon 24341, Korea; dr10126@naver.com (M.C.S.); cjhemd@kangwon.ac.kr (J.H.C.); 7 Department of Anesthesiology and Pain Medicine, Chungju Hospital, Konkuk University School of Medicine, Chungju, Chungbuk 27376, Korea; leekyu@kku.ac.kr

**Keywords:** anti-epileptic drug, transient cerebral ischemia, pyramidal neurons, neuroprotection, glial activation

## Abstract

Oxcarbazepine, an antiepileptic drug, has been reported to modulate voltage-dependent sodium channels, and it is commonly used in epilepsy treatment. In this study, we investigated the neuroprotective effect of oxcarbazepine in the hippocampus after transient ischemia in gerbils. Gerbils randomly received oxcarbazepine 100 or 200 mg/kg before and after transient ischemia. We examined its neuroprotective effect in the cornu ammonis 1 subfield of the gerbil hippocampus at 5 days after transient ischemia by using cresyl violet staining, neuronal nuclei immunohistochemistry and Fluoro-Jade B histofluorescence staining for neuroprotection, and by using glial fibrillary protein and ionized calcium-binding adapter molecule 1 immunohistochemistry for reaction of astrocytes and microglia, respectively. Pre- and post-treatment with 200 mg/kg of oxcarbazepine, but not 100 mg/kg of oxcarbazepine, protected pyramidal neurons of the cornu ammonis 1 subfield from transient ischemic damage. In addition, pre- and post-treatment with oxcarbazepine (200 mg/kg) significantly ameliorated astrocytes and microglia activation in the ischemic cornu ammonis 1 subfield. In brief, our current results indicate that post-treatment as well as pre-treatment with 200 mg/kg of oxcarbazepine can protect neurons from ischemic insults via attenuation of the glia reaction.

## 1. Introduction

Transient global cerebral ischemia (TGCI) caused by a brief interruption of blood supply to the brain can develop the death or loss of neurons in vulnerable brain regions such as cornu ammonis 1 subfield (CA1) of the hippocampus [1]. Pyramidal neurons in the hippocampal CA1, which are called CA1 pyramidal neurons, are vulnerable to TGCI [2,3]. Neuronal death/loss in CA1 occurs a few days after TGCI, thus, it is named “delayed neuronal death (DND)” [4,5]. In addition, the CA1 neuronal loss is related to locomotor hyperactivity after TGCI in gerbils [6,7]. It has been known that the mechanisms of the death of the CA1 pyramidal neurons following TGCI include excitotoxicity by glutamate toxicity, oxidative stress via reactive oxygen species (ROS), neuroinflammation by glia cells, etc. [8,9,10]. 

Various antiepileptic drugs (AEDs) have been studied to play some useful effects beyond their original effectiveness [11,12,13,14]. For example, AEDs displays beneficial effects to counteract neuronal damage or death from experimentally induced brain injuries, such as ischemic stroke, intracerebral hemorrhage and trauma [15]. Mechanisms of AEDs have been suggested four effects at the synaptic level: (i) regulation of voltage-dependent sodium channels, (ii) regulation of voltage-dependent calcium channels, (ii) improvement of GABA-mediated neuronal inhibition and (iv) decrease of glutamate mediated excitatory transmission [16]

Among AEDs, oxcarbazepine (OXC), which had been made from a beneficial alternative to carbamazepine (10,11-keto analog of carbamazepine) [17], is one of anticonvulsant compounds commonly used in epilepsy treatment [18] through inhibition of voltage-dependent sodium channels [19]. Voltage-gated sodium channel in the nervous system plays significant roles in initiation and propagation of the action potential as well as in regulation of pathophysiological steps during initial ischemic stage, suggesting that sodium-channel modulating drugs can play a neuroprotective role [20,21].

We recently demonstrated that lacosamide, a voltage-gated sodium channel related AED, exerted neuroprotective effects against transient cerebral ischemic damage in gerbils [22]. In addition, it has been reported that OXC shows neuroprotective effect in an in vitro model of ischemia [23]. However, the effects of OXC in an in vivo model of ischemia are unclear. In this regard, we examined neuroprotective effect of OXC in the hippocampus in a gerbil model of TGCI. In addition, we also examined effects of OXC on glial activation in the gerbil ischemic hippocampus.

## 2. Materials and Methods

### 2.1. Experimental Animals

Mongolian gerbils *(Meriones unguiculatus*) were supplied from the Experimental Animal Center, Hallym University, Chuncheon, Republic of Korea and used at about 6 months (body weight, 65–75 g) of age. The gerbils were housed in a conventional state maintained at temperature (about 23 °C) and humidity (about 60%) control with a 12-h light/dark cycle. They were given ad libitum access to tap water and commercial food. All experimental procedures including handling and caring of animals were performed in accordance with current international laws and policies (Guide for the Care and Use of Laboratory Animals. 8th edition) [24]. In this study, we minimized numbers of gerbils and their suffering as much as possible.

### 2.2. Treatment with OXC

In order to prove neuroprotective effects of OXC against ischemic damage after ischemia–reperfusion, a total of 56 gerbils were used and divided into the following eight groups (*n* = 7 in each group): two sham groups (1 and 2), which were pre- and post-treated with vehicle (saline) before and after TGCI and subjected to sham TGCI; two ischemia groups (3 and 4), which were pre- and post-treated with vehicle (saline) before and after TGCI and subjected to TGCI; two 100 mg/kg OXC ischemia groups (5 and 6), which were pre- and post-treated with 100 mg/kg OXC before and after TGCI and subjected to TGCI; two 200 mg/kg OXC ischemia groups (7 and 8), which were pre- and post-treated with 200 mg/kg OXC before and after TGCI and subjected to TGCI. The results of group 1 and 2 and group 3 and 4 were very similar, and we presented only the results of group 1 and 3 in this study. 

OXC was dissolved in 10% Tween 80 (in saline) and administered intraperitoneally three times daily for 3 days prior to TGCI or immediately after TGCI.

### 2.3. Induction of TGCI

Induction of TGCI in gerbils was done according to method described in our published paper [25]. All gerbils were initially anesthetized by using of 2.5% isoflurane (Baxter, Deerfield, IL, USA) in a N2O (67%) and O2 (33%) mixture (*v*/*v*) via facemask. Anesthesia was maintained with 2% isoflurane. Briefly, TGCI in the gerbils was achieved by occlusion of both common carotid arteries. Namely, the arteries were isolated in the neck and occluded of 5 min by using non-traumatic aneurysm clips (Yasargil FE 723K, Aesculap, Tuttlingen, Germany). The complete occlusion of blood flow was determined by observing no circulation in retinal central arteries with an ophthalmoscope (HEINE K180®, Heine Optotechnik, Herrsching, Germany). The aneurysm clips were released from the common carotid arteries after 5-min occlusion. The gerbils were controlled under normothermic conditions (37 ± 0.5 °C) during and after TGCI operation and until euthanasia. The gerbils of the sham groups received the same surgical procedures without both common carotid artery occlusion. 

### 2.4. Tissue Preparation for Histology 

For histological examination, the sham or ischemic gerbils were anesthetized by intraperitoneal injection of sodium pentobarbital (60 mg/kg) (JW Pharm. Co., Ltd., Seoul, Korea). The gerbils were perfused transcardially with 0.1 M phosphate-buffered saline (pH 7.4) to remove blood, and their brains were fixed by perfusion of 4% paraformaldehyde in 0.1 M phosphate buffer (pH 7.4). Subsequently, their brains were removed from the skulls and post-fixed by immersion in the same fixative for 5 h. The brain tissues were infiltrated with 30% sucrose overnight to protect the brains from damage during section. The cryoprotected brain tissues were frontally and serially cut (30-μm thickness) in a cryostat (Leica, Wetzlar, Germany), and they were stored into 12-well plates for histological examination.

### 2.5. Cresyl Violet (CV) Staining 

In order to examine morphological change or damage in ischemic hippocampi induced by TGCI, CV staining was done as we described previously [25]. In brief, CV acetate (Sigma-Aldrich, St. Louis, MO, USA) was dissolved at 1.0% (*w*/*v*) in distilled water (DW), and glacial acetic acid (GAA) was added to this solution until GAA was 0.28%. The sections were stained with CV solution, and they were prepared as permanent slides for examination under a microscope.

### 2.6. Fluoro-Jade B (FJB) Histofluorescence Staining

To examine neuronal degeneration, histofluorescence staining with FJB (a reliable fluorescent marker for the localization of degeneration) (Histochem, Jefferson, AR, USA) was performed according to our published procedure [26,27]. In brief, the prepared sections were immersed in 1% sodium hydroxide solution dissolved in 80% ethanol, and the immersed sections were transferred to 0.06% potassium permanganate solution. They were then incubated in 0.0004% FJB solution, washed with PBS (pH 7.4) and set on a slide warmer (approximately 50 °C) for FJB reaction. Finally, the reacted sections were prepared for examination under an epifluorescent microscope.

### 2.7. Immunohistochemistry

In order to study neuronal damage and glial activation in the ischemic hippocampi, immunohistochemistry was performed according to our published protocol [27,28]. We used antibodies as follows: (1) neuronal nuclei (NeuN) for neurons; (2) glial fibrillary acidic protein (GFAP) for astrocytes; (3) ionized calcium-binding adapter molecule 1 (Iba-1) for microglia. In brief, the brain sections were treated with 0.3% hydrogen peroxide (H_2_O_2_) in PBS (pH 7.4) for 40 min, followed by 10% normal rabbit serum (Vector Laboratories, Inc., Burlingame, CA, USA) in PBS for 40 min. Furthermore, these pretreated sections were reacted with mouse anti-NeuN (diluted 1:1000) (Chemicon, Temecula, CA, USA) for neurons, mouse anti-GFAP (diluted 1:800) (Chemicon, Temecula, CA, USA) for astrocytes and rabbit anti-Iba-1 (diluted 1:800) (Chemicon, USA) for microglia. Subsequently, these sections were exposed to biotinylated goat anti-mouse immunoglobulin G (IgG) or goat anti-rabbit IgG and streptavidin peroxidase complex (diluted 1:200, respectively) (Vector, Burlingame, CA, USA), and they were visualized with 3,3’-diaminobenzidine (in 0.1 M Tris HCl buffer (pH 7.4). Finally, the immunoreacted sections were prepared for examination of each immunoreactivity under a light microscope.

### 2.8. Data Analysis

The studied tissue sections were selected according to anatomical landmarks corresponding to Bregma −1.65 mm ~ −3.00 mm of the gerbil brain atlas [29]. To appraise the neuroprotective effect of OXC, numbers of NeuN-immunoreactive and FJB-positive cells were counted in the hippocampal CA1 according to our previous method [25]. Briefly, digital images from seven sections per animal, which were selected with a 120-µm interval, were captured with an AxioM1 light microscope (Carl Zeiss, Germany) for NeuN-immunoreactive neurons or an epifluorescent microscope (Carl Zeiss, Göttingen, Germany) with blue (450–490 nm) excitation light and a barrier filter for FJB-positive cells. The microscopes were equipped with digital camera (Axiocam, Carl Zeiss, Germany) connected to a PC monitor. Cells were captured in a 250 × 250 μm square including the stratum pyramidale at the center of the CA1, and the number of cells were analyzed by using an image analyzing system (software) (Optimas 6.5, CyberMetrics, Scottsdale, AZ, USA). Cell counts were obtained by averaging the counts from all animals per group.

To quantitatively analyze the density of GFAP and Iba-1-immunoreactive structures in the CA1, images of these structures were captured through the same method described above. Firstly, densities of GFAP- and Iba-1-immunoreactive structures were evaluated based on an optical density (OD), which was obtained after transformation of the mean gray level of the immunoreactivity by using a formula: OD = log (256/mean gray level). The background of each immunoreactivity was subtracted, and a ratio of the OD of image file was calibrated into relative % as a relative optical density (ROD) by using Adobe Photoshop (version 8.0) and NIH Image J software (National Institutes of Health, Bethesda, MD, USA). The ROD of each immunoreactive structure in each group was calibrated as % of the sham group (100%).

### 2.9. Statistical Analysis

The data shown here represent the means ± standard error of the mean (SEM). Differences of the means among the groups were statistically analyzed by two-way analysis of variance (ANOVA) with a post hoc Bonferroni’s multiple comparison test in order to elucidate the effects of OXC. Statistical significance was considered at *p* < 0.05.

## 3. Results

### 3.1. Neuroprotection

#### 3.1.1. CV Staining

**Figure 1:**
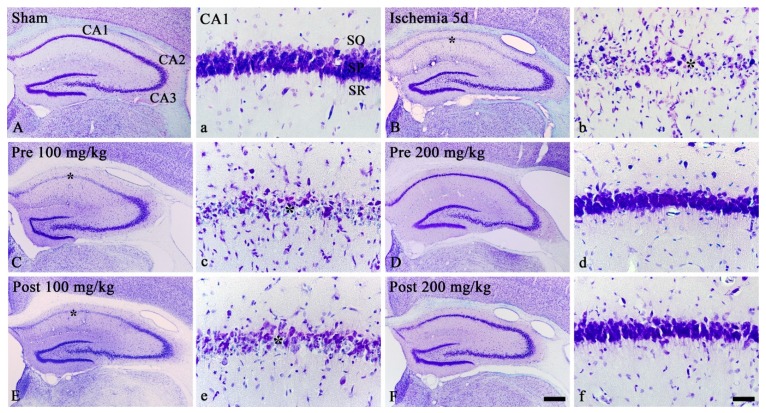
CV staining in the gerbil hippocampus of the sham (**A**,**a**), ischemia (**B**,**b**), OXC pretreated ischemia (**C**,**c**,**D**,**d**) and OXC posttreated ischemia (**E**,**e**,**F**,**f**) groups at 5 days after TGCI. In the ischemia and both 100 mg/kg OXC treated ischemia groups, most of CV-positive cells in the stratum pyramidale (SP) (asterisks) of the CA1 region are damaged or lost. However, in both 200 mg/kg OXC treated ischemia groups, CV-positive cells are not damaged. CA, cornu ammonis; CV, cresyl Violet; OXC, oxcarbazepine; SO, stratum oriens; SR, stratum radiatum; TGCI, transient global cerebral ischemia. Scale bars = 400 μm (**A,B,C,D,E,F**) and 40 μm (**a,b,c,d,e,f**).

We examined morphological changes in all cells in the sham and ischemic hippocampi by staining with CV, which is used for Nissl’s body. In the sham groups, CV staining showed all cells which were located in all layers: in particular, large CV-positive cells formed the stratum pyramidale (SP) in the hippocampus proper, which consisted of CA1-3 (Figure 1A,a). In the ischemia groups, most of CV-positive cells in the SP were damaged or lost at 5 days after TGCI, showing that small CV-positive cells were apparently increased in numbers in all layers (Figure 1B,b). 

In the ischemia groups pre- and post-treated with 100 mg/kg OXC, the distribution pattern of CV-positive cells at 5 days postischemia was similar to that in the ischemia-groups (Figure 1C,c,E,e). However, in the ischemia groups pre- and post-treated with 200 mg/kg OXC, CV-positive cells in the SP were protected from ischemic injury, showing that the distribution pattern of CV-positive cells in these groups was similar to that in the sham groups (Figure 1D,d,F,f).

#### 3.1.2. NeuN Immunohistochemistry

**Figure 2:**
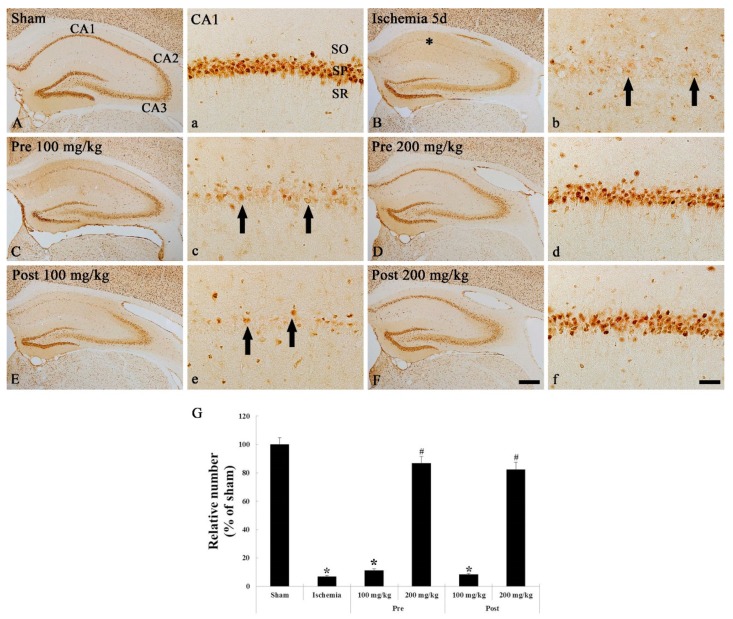
NeuN immunohistochemistry in the hippocampus of the sham (**A,a**), ischemia (**B,b**), OXC pretreated ischemia (**C,c,D,d**) and OXC posttreated ischemia (**E,e,F,f**) groups at 5 days after TGCI. Most of NeuN-positive neurons are lost in the stratum pyramidale (SP) (arrows) of the CA1 region of the ischemia and both 100 mg/kg OXC treated ischemia groups. However, in both 200 mg/kg OXC treated ischemia groups, many NeuN-immunoreactive neurons (asterisks) are observed in the SP of the CA1 region. CA, cornu ammonis; NeuN, neuronal nuclei; OXC, oxcarbazepine; SO, stratum oriens; SR, stratum radiatum; TGCI, transient global cerebral ischemia. Scale bar = 400 μm (**A,B,C,D,E,F**) and 40 μm (**a,b,c,d,e,f**). (G) The mean number of NeuN-immunoreactive neurons in the SP of the CA1 region at 5 days after TGCI (*n* = 7 in each group, * *p* < 0.05, significantly different from the sham group, ^#^ *p* < 0.05, significantly different from the ischemia group). The bars indicate the means ± standard error of the mean (SEM).

We examined neuronal changes in the sham and ischemic hippocampi by immunohistochemical staining with NeuN, which is a neuronal nuclear antigen that is commonly used as a biomarker for neurons. In the sham groups, NeuN-immunoreactive neurons were mainly shown in the SP of the hippocampus (Figure 2A,a). In the ischemia groups, numbers of NeuN-immunoreactive neurons of the SP were significantly reduced (8.7% of the sham group) compared with the sham group at 5 days after TGCI (Figure 2B,b,G). 

In the 100 mg/kg OXC pre- and post-treated ischemia groups, findings of NeuN immunohistochemistry were similar to those in the ischemia groups: a few NeuN-immunoreactive neurons were shown in the SP of the CA1 region (Figure 2C,c,E,e,G). However, the number of NeuN-immunoreactive neurons in the SP of the CA1 region of the 200 mg/kg OXC pre- and post-treated ischemia groups was similar (88.2% and 86.1%, respectively, of the sham groups) to that in the sham groups, and the distribution pattern of NeuN-immunoreactive neurons was also similar to that in the sham groups (Figure 2D,d,F,f,G).

#### 3.1.3. FJB Histofluorescence Staining

**Figure 3:**
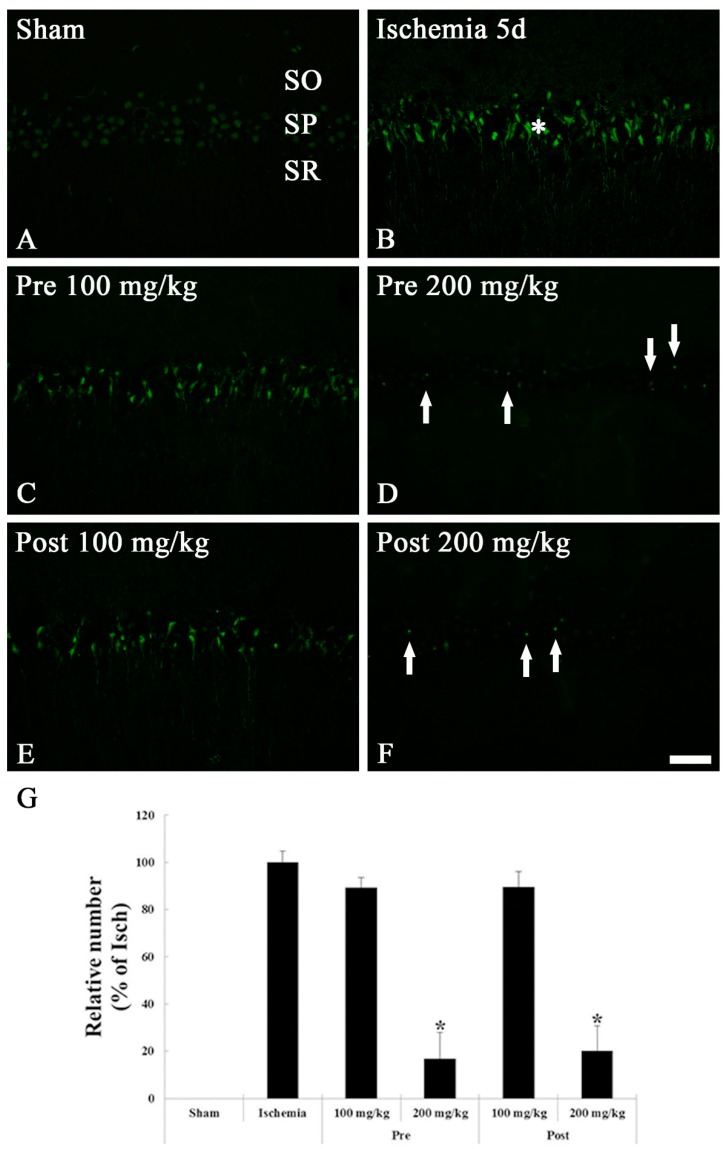
FJB histofluorescence staining in the CA1 region of the sham (**A**), ischemia (**B**), OXC pretreated ischemia (**C**,**D**) and OXC posttreated ischemia (**E**,**F**) groups at 5 days after TGCI. In the ischemia and both 100 mg/kg OXC pre- and post-treated ischemia groups, many FJB-positive cells are found in the stratum pyramidale (SP) (asterisks), while FJB-positive cells are rarely detected in the SP (arrows) of the 200 mg/kg OXC pre- and post-treated ischemia groups. CA, cornu ammonis; FJB, Fluoro Jade B; OXC, oxcarbazepine; SO, stratum oriens; SR, stratum radiatum; TGCI, transient global cerebral ischemia. Scale bar = 40 μm. (G) The mean number of FJB-positive cells in the SP of the CA1 region at 5 days after TGCI (* *p* < 0.05, significantly different from the ischemia group). The bars indicate the means ± standard error of the mean (SEM).

We examined cellular degeneration/death in the sham and ischemic hippocampi by histofluorescence staining with FJB, which is a marker for location of cellular degeneration. FJB-positive cells were not shown in the SP of the CA1 region of the sham groups (Figure 3A). In the ischemia groups, many FJB-positive cells with color of jade were detected in the SP at 5 days after TGCI (Figure 3B,G). 

In both 100 mg/kg OXC treated ischemia groups, a large number of FJB-positive cells (89.6% and 84.0% of the ischemia groups, respectively) were distributed in the SP of the CA1 region as in the ischemia groups (Figure 3C,E,G). However, we found a few FJB-positive cells (8.2% and 10.1% of the ischemia groups, respectively) in the SP of the 200 mg/kg OXC pre- and post-treated ischemia groups 5 days after TGCI, showing that neurons in the SP were protected from ischemic injury by pre- or post-treatment with 200 mg/kg OXC (Figure 3D,F,G).

### 3.2. Glial Activation 

#### 3.2.1. GFAP Immunohistochemistry

**Figure 4:**
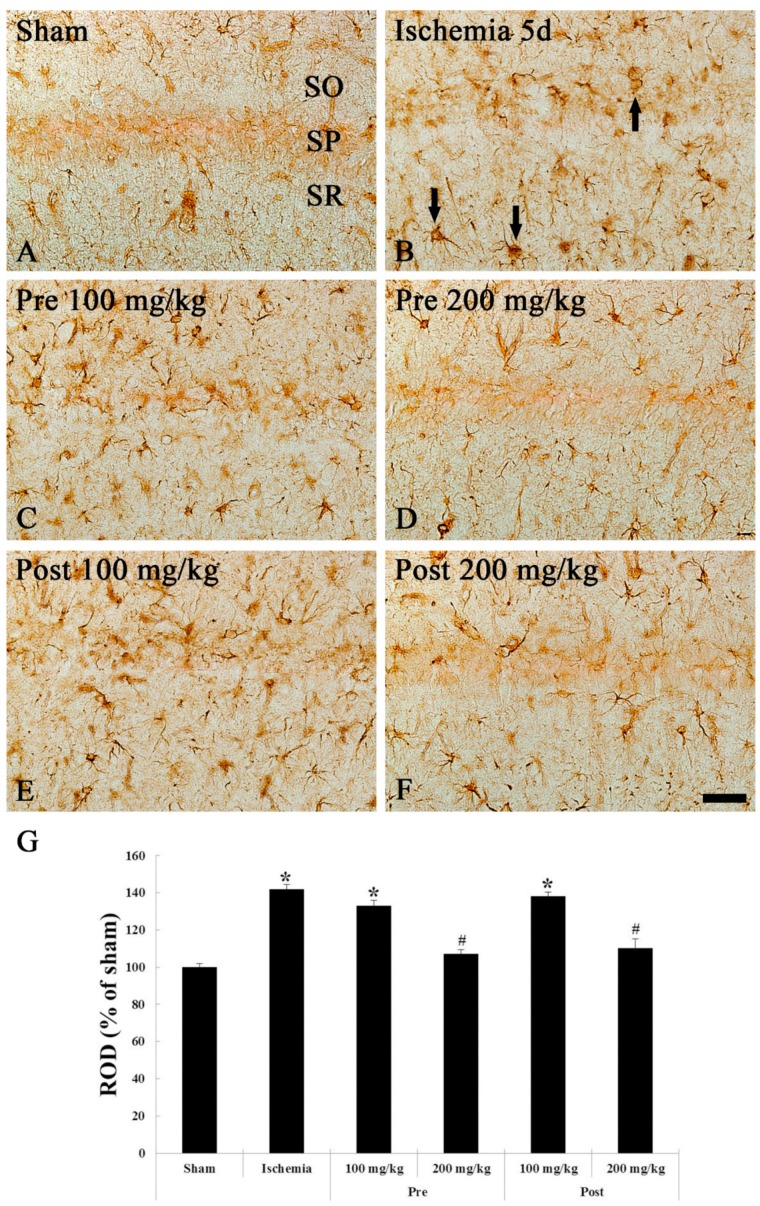
GFAP immunohistochemistry in the CA1 region of the sham (**A**), ischemia (**B**), OXC pretreated ischemia (**C**,**D**) and OXC posttreated ischemia (**E**,**F**) groups at 5 days after TGCI. GFAP-immunoreactive astrocytes (arrows) are markedly activated (hypertrophied cytoplasm and processes) in the ischemia and both 100 mg/kg OXC pre- and post-treated ischemia groups. However, GFAP immunoreactivity in both 200 mg/kg OXC ischemia groups is significantly lower than that in the ischemia- group. CA, cornu ammonis; GFAP, glial fibrillary acidic protein; OXC, oxcarbazepine; SO, stratum oriens; SP, stratum pyramidale; SR, stratum radiatum; TGCI, transient global cerebral ischemia. Scale bar = 40 μm. (G) Relative optical density (ROD) as percentage values of the GFAP immunoreactivity at 5 days after TGCI (* *p* < 0.05, significantly different from the sham group, # *p* < 0.05, significantly different from the ischemia group). The bars indicate the means ± standard error of the mean (SEM).

Immunohistochemistry with GFAP is used to detect astrocytes as a biomarker for astrocytes. At 5 days after sham TGCI, GFAP-immunoreactive astrocytes in the CA1 region of the sham groups were scattered in all layers as resting form of astrocytes with small cytoplasm and thread-like processes (Figure 4A). However, at 5 days after TGCI in the ischemia groups, GFAP-immunoreactive astrocytes were activated (altered), namely, they had a bulky cytoplasm with thickened processes as reactive astrocyte form (Figure 4B). In this group, the ROD, meaning the density of GFAP-immunoreactive structures (cell bodies and processes of astrocytes), was significantly increased (141.2% of the sham group) in the CA1 region compared with that in the sham group (Figure 4G). 

At 5 days post-TGCI in the 100 mg/kg OXC pre- and post-treated ischemia groups, morphological characteristics of GFAP-immunoreactive astrocytes was similar to that in the ischemia groups (Figure 4C,E). On the other hand, distribution and morphological characteristics of GFAP-immunoreactive astrocytes in both pre- and post-200 mg/kg OXC treated groups were similar to the sham groups (Figure 4D,F), showing that the ROD was decreased by 18.7% and 34.3%, respectively, compared to that in the pre- and post-100 mg/kg OXC treated group (Figure 4G).

#### 3.2.2. Iba-1 Immunohistochemistry

**Figure 5:**
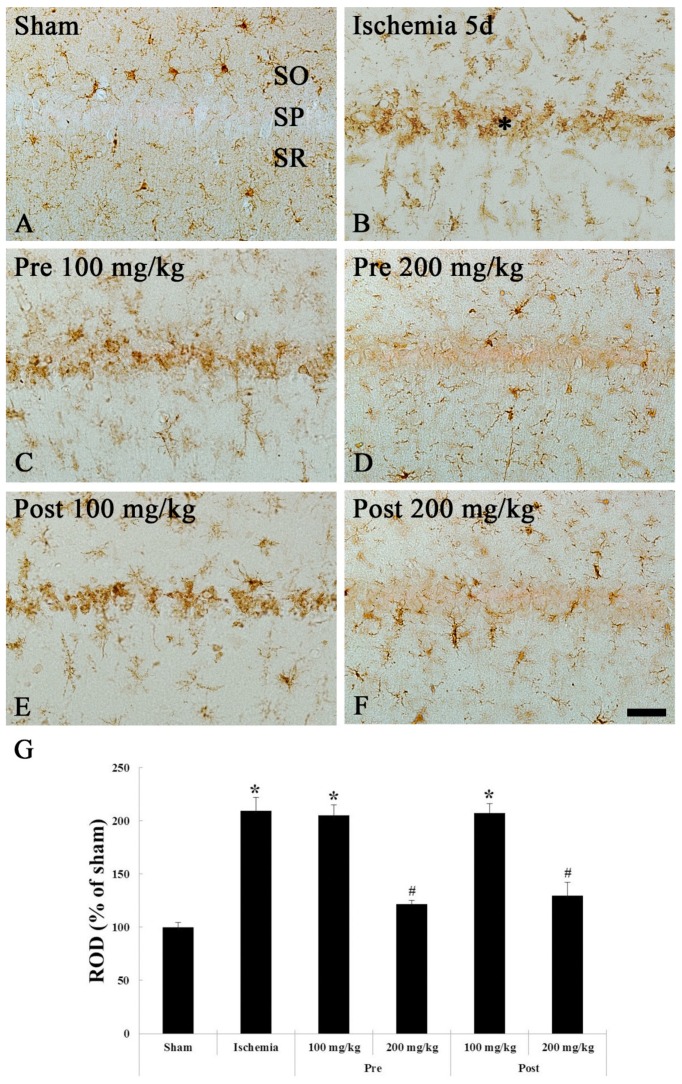
Iba-1 immunohistochemistry in the CA1 region of the sham (**A**), ischemia (**B**), OXC pretreated ischemia (**C**,**D**) and OXC posttreated ischemia (**E**,**F**) groups at 5 days after TGCI. Iba-1-immunoreactive microglia are markedly activated (hypertrophied cytoplasm and processes) and aggregate within the stratum pyramidale (SP) (asterisk) in the ischemia and both 100 mg/kg OXC ischemia groups. However, in both 200 mg/kg OXC ischemia groups, Iba-1-immunoreactive microglia are similar to those in the sham group. CA, cornu ammonis; Iba-1, ionized calcium binding adaptor molecule 1; OXC, oxcarbazepine; SO, stratum oriens; SR, stratum radiatum; TGCI, transient global cerebral ischemia. Scale bar = 40 μm. (**G**) ROD as percent values of the Iba-1 immunoreactivity at 5 days after TGCI (* *p* < 0.05, significantly different from the sham group, # *p* < 0.05, significantly different from the ischemia group). The bars indicate the means ± SEM.

Immunohistochemistry with Iba-1 is used to detect microglia (microglial cells) as a biomarker for microglia. In the sham groups, Iba-1-immunoreactive microglia as resting (ramified) microglia were distributed in all layers in the CA1 region, showing that they had small cytoplasm and thin branched processes at 5 days after sham TGCI (Figure 5A). In the ischemia groups, Iba-1-immunoreactive microglia were markedly activated, showing that they had a hypertrophied cell body, short and thickened processes (Figure 5B). In addition, many of them congregated in or near the SP of the CA1 region (Figure 5B). In this group, the ROD was increased by 101.5% compared to that in the sham group (Figure 5G). 

In the 100 mg/kg OXC pre- and post-treated ischemia groups, the distribution, morphology and ROD of Iba-1-immunoreactive microglia were similar to those in the ischemia groups (Figure 5C,E,G). However, in the 200 mg/kg OXC pre- and post-treated ischemia groups, the activation of Iba-1-immunoreactive microglia was significantly alleviated at 5 days after TGCI, showing that the ROD was significantly decreased by 76.1% and 62.2%, respectively, compared to that in the pre- and post- 100 mg/kg OXC treated group (Figure 5D,F,G). 

## 4. Discussion

To the best of our knowledge, our current study is the first to have demonstrated a neuroprotective effect of OCX in the hippocampus in a gerbil model of transient cerebral ischemia. The DND in the CA1 hippocampal neurons after TGCI has been reported to be the apoptosis [30], necroptosis [31], necrosis [32,33] and autophagy [34]. We examined neuroprotective effect of pre- and post-treatment with 100 and 200 mg/kg OXC against damage by TGCI in the gerbil hippocampal CA1 region 5 days after 5-min TGCI by using NeuN immunohistochemistry and FJB histofluorescence staining (a useful marker for all types of neurodegeneration), and we found that both pre- and post-treatment with 200 mg/kg OXC, but not 100 mg/kg OXC, protected pyramidal neurons of the CA1 region from TGCI. These results indicate that OXC showed both preventive and protective effects against ischemic neuronal death in the hippocampus after TGCI. It has been suggested that at least three dose levels are usually adequate in order to establish an optimal experimental design suitable for dose-response studies [35,36]. In this study, the limitation was that OXC efficacy in TGCI was confirmed only at two dose levels to reduce the number of animals. In future studies, it is necessary to examine the efficacy of OXC between 100 mg/kg and 200 mg/kg. 

Previous studies have suggested that cerebral ischemia is closely associated with inflammatory reactions, which are related to a break of the balance between pro- and anti-inflammatory cytokines [37,38]. Together, it is well known that cerebral ischemia leads to activation of astrocytes and microglia, and the activated glial cells commonly produce and release numerous cytokines that are involved with ischemia-induced neuronal damage or death [39,40,41]. Therefore, we think it is necessary to inhibit glial cell activation for the neuroprotective effect against cerebral ischemic injuries. 

In this study, pre- and post-treatment with 200 mg/kg OXC significantly attenuated the activation of astrocytes and microglia in the CA1 region of the hippocampus compared with that in the ischemia groups following 5 mins of TGCI. OXC is a recently developed analogue in an attempt to maintain benefits of Carbamazepine (CBZ). It has been reported that CBZ has a capacity to inhibit microglial activity in inflammatory penumbra in myelin-specific T cell receptor transgenic mice [42]. In addition, CBZ attenuates expressions of tumor necrosis factor alpha (TNF-a) and interleukin (IL)-1b and reduces increases of pro-inflammatory cytokines in the hippocampus after lipopolysaccharide injection in rats [43]. Therefore, in the present study, the attenuation of ischemia-induced glial cells activation in the hippocampal CA1 region by treatment of OXC might be associated with the neuroprotective effect against damage by TGCI.

In the future, we have to study other mechanisms of OXC in neuroprotection against ischemic stroke. It has have been reported that AEDs have beneficial therapeutic effects through multiple mechanisms in brain diseases, including cerebral ischemia (ischemic stroke), epilepsy and multiple sclerosis and classical neurodegenerative diseases including Alzheimer’s disease and Parkinson’s disease [44,45,46,47]. Mechanisms of AEDs are as follows: regulation of the activation of voltage-gated ion channels, promotion of gamma-aminobutyric acid (GABA)-mediated inhibitory neurotransmission, decrease of glutamate-mediated excitatory neurotransmission, etc. [48,49]. Among the mechanisms of AEDs, the main mechanism of OXC is known to play inhibition of voltage-dependent sodium channels [19]. Recently, it was demonstrated that OXC exerted beneficial effects through preventing exocytotic glutamate release and altering recurrent depolarization [50]. In addition, OXC reduced Ca^2+^ influx through block of pre- or post-synaptic Ca^2+^ channels in the CA1 area of rat hippocampal slices [51]. However, protective effects of OXC against damage induced by cerebral ischemic insults have not been investigated yet. 

It has been demonstrated that pre-treatment with OXC type AEDs including CBZ, Felbamate and Lamotrigine exerted neuroprotective effects by inhibition of voltage-sensitive Na^+^ channels in animal models of stroke [13] and neurodegenerative diseases [41]. In a mouse model of seizure-induced neuronal injury, Park et al. (2015) reported that CBZ showed neuroprotection against KA-induced neurotoxicity through increased phosphorylation of the signal transducer and activation of transcription-3 (Stat3) [52]. In animal models of brain ischemia, it was reported that Felbamate treatment prevented selective neuronal loss in the hippocampal CA1 region in a gerbil model of global ischemia [53] and a rat model of hypoxia-ischemia [54]. 

## 5. Conclusions

In conclusion, the results of our present study showed that pre- and post-treatment with 200 mg/kg OXC protected neuronal cell death/damage in the gerbil hippocampus induced by TGCI and significantly attenuated glial cell activation in the ischemic hippocampus. These findings indicate that pre- and post-treatment with 200 mg/kg OXC is able to use for protection of neuronal cell death/damage form ischemic insults. However, more studies need to be done on other mechanisms of OXC in neuroprotection from ischemic stroke.
